# An evaluation of virtual supervision effectiveness within department of veterans affairs (VA) health professionals training programs

**DOI:** 10.1186/s12909-025-08062-1

**Published:** 2025-10-24

**Authors:** Linda M. Kawentel, John P. Donnelly, Kaylee W. Burgan, Gezan M. Yahya, Joanna S. Doredla, Jessica L. Johnson, Kimberly Falco, Marjorie A. Bowman, John Byrne, Nicholas W. Bowersox

**Affiliations:** 1 VA QUERI Center for Evaluation and Implementation Resources (CEIR), Veterans Health Administration, Ann Arbor, MI USA; 2 Center for Clinical Management Research (CCMR), Veterans Health Administration, Ann Arbor, MI USA; 3 Department of Learning Health Sciences, University of Michigan, Ann Arbor, MI USA; 4 VA Office of Academic Affiliations, Veterans Health Administration, Washington, D.C, USA; 5 School of Nursing, Nevada State University, Henderson, NV USA; 6 Department of Family Medicine and Community Health, University of Pennsylvania, Philadelphia, PA USA; 7 Department of Psychiatry and Department of Learning Health Sciences, University of Michigan, Ann Arbor, MI USA

**Keywords:** Telesupervision, Virtual training, Health professions education, Veterans, Program evaluation

## Abstract

**Background:**

Limited information exists on whether virtual training is equivalent to traditional in-person training in supporting the development of clinical providers.

**Methods:**

A multi-site evaluation using survey methods was conducted with a multidisciplinary group of health professions trainees and supervisors within the Veterans Health Administration to assess the equivalence of two supervision modalities – exclusively in-person supervision and supervision incorporating at least some virtual supervision – on trainee clinical competency development, trainee preparedness to respond to patient emergencies, and effective trainee/supervisor working relationships.

**Results:**

Trainees who experienced some virtual supervision rated their clinical competency levels as higher than trainees with only in-person supervision on competencies related to patient care, knowledge for practice, interpersonal and communication skills, practice-based learning and improvement, and systems-based practice. This trainee group also rated their level of preparation to respond to patient emergencies and several aspects of their supervisory working relationship more highly than in-person only trainees. Compared to those providing only in-person supervision, supervisors conducting some virtual supervision also rated their trainees as having higher levels of clinical competency on patient care, practice-based learning and improvement, and systems-based practice, as well as higher preparedness to respond to patient emergencies. Challenges and benefits to virtual supervision were also noted, though most trainees and supervisors who had participated in virtual supervision held a positive view of this modality.

**Conclusions:**

These data constitute the first evaluation of the equivalency of some virtual and in-person only supervision in supporting trainee skill development and supervisory working relationships based on feedback from trainees and supervisors across multiple clinical disciplines.

**Trial registration:**

Not applicable.

**Supplementary Information:**

The online version contains supplementary material available at 10.1186/s12909-025-08062-1.

## Background

The COVID-19 pandemic led to a focus on trainee/supervisor contact and training via virtual interaction modalities, or virtual supervision [1, 2]. Virtual supervision appears to offer benefits relative to traditional in-person supervision in terms of time and efficiency [3–5] and has been implemented at scale within some large training settings, such as the U.S. Veterans Affairs (VA) Health Administration, with this implementation being well-received by trainees across multiple clinical disciplines [6]. It also has the potential to expand training offerings to rural areas, where telecommunications technology can be used to link trainees to supervisors with clinical expertise which may not be available in the local clinical setting [7, 8]. However, limited available information exists on whether virtual supervision is adequate to meet the development needs of trainees, including clinical competency development, effective supervisory working relationships, and professional development skills.

Current scholarship on the equivalency of in-person and virtual supervision has largely focused on the supervisory working-alliance within small samples of psychology and counseling trainees [4, 9–14]. For example, Inman et al. [9] found that more than half of counseling and psychology doctoral students reported that in-person and virtual supervision approaches contributed equally to strong supervisory relationships. Likewise, Tarlow et al. [15] found that, in a small sample of psychology trainees who switched from an initial in-person supervision format to exclusively virtual supervision, the two forms of supervision were rated as equivalent on outcomes concerning participant supervision satisfaction and the supervisory working alliance. Similarly, Jordan and Shearer [10] found that VA psychology trainees in rotations involving in-person supervision did not differ from those in virtual supervision rotations in how they rated the quality of their supervisor–supervisee relationships though, interestingly, rapport with one’s supervisor was higher among those trainees who received virtual supervision.

Given the growth of telemedicine and virtual supervision, training leaders in the health professions need to understand the effectiveness of virtual approaches to supervision, compared to traditional in-person supervision, beyond the counseling and psychology disciplines. Moreover, more studies of virtual supervision are needed with larger sample sizes of both trainees and supervisors. As the largest training program for health professions trainees in the United States, the Veterans Health Administration is both vested in expanding high quality care for America’s Veterans and providing advanced training opportunities for health professions trainees. A recent survey found that approximately one-fourth of VA trainees reported participating in virtual supervision, with statistically significant differences by profession [6]. VA needs clear information regarding the value of virtual supervision in supporting clinical trainee development and ensuring effective, safe, and appropriate care to Veteran patients. To respond to this need, an evaluation was conducted in collaboration with VA’s Office of Academic Affiliations (OAA), which oversees VA’s Health Professions Education (HPE) training programs, which focused on the following key questions:

1. Is virtual supervision as effective as in-person supervision to support skill development and trainee/supervisor working relationships for VA trainees and supervisors?
2. What are trainee and supervisor perspectives on the use of virtual supervision within VA?

## Methods

### Evaluation design

Survey methods were utilized to assess the equivalence of some virtual supervision to exclusively in-person supervision in terms of supporting trainee competency development, response to patient emergencies, establishment of effective trainee/supervisor working relationships, and perspectives on the contributions and challenges of virtual supervision in VA HPE training programs.

The evaluation was guided by an adapted version of the CDC’s Framework for Program Evaluation in Public Health [16] which was used to inform evaluation processes and facilitate collaboration between OAA and the evaluation team.

### Sampling

The surveys were distributed to trainees and supervisors working in VA Primary Care and Outpatient Mental Health (OPMH) clinical settings. These settings were selected because of the trainee large volume and consistent core clinical duties. Trainees and supervisors from the following disciplines were targeted for participation: (1) Family Medicine, (2) Internal Medicine, (3) Psychiatry, (4) Psychology, and (5) Nursing (specifically Nurse Practitioner). Disciplines were selected based on guidance from OAA, as well as their higher rates of telehealth care delivery within the clinical settings of interest.

Information on telehealth usage rates in Primary Care and OPMH was generated from available administrative data within the VA’s Corporate Data Warehouse and used as a proxy for virtual supervision use at VA sites. To capture a diversity of supervision experiences, sites were targeted based on the highest and lowest quartiles of telehealth usage in Primary Care and OPMH, calculated at each level of site complexity [17, 18] to ensure of range of experiences with the provision of telehealth. Sites were selected to represent each possible combination of these factors: site complexity (1 A-3), telehealth delivery in Primary Care (first quartile/fourth quartile), telehealth delivery in outpatient Mental Health (first quartile/fourth quartile). All possible combinations of these factors resulted in 20 potential site profiles which were then used to target sites for inclusion in this evaluation. When a selected site declined participation, another site from the targeted profile was selected. If no eligible sites remained, sites that fit the next closest quartile (e.g., replacing the 4th quartile with the 3rd quartile) were contacted. In total, 20 sites agreed to participate in the evaluation.

### Survey instruments

Similar survey instruments were developed for completion by trainees (supplemental file 1) and supervisors (supplemental file 2) which included questions related to the structure and format of virtual supervision. Supervision questions focused on events that occurred during an episode of care as well as other activities that supported care delivery but took place outside of direct care delivery (e.g., review of procedures, didactic teaching, mentoring). Additional questions assessed the perceived quality of training and supervision experience, trainee/supervisor relationship, perceptions of skill acquisition, virtual disruptions, and attitudes regarding virtual supervision. Outcomes assessing clinical competency areas were adapted from the Accreditation Council for Graduate Medical Education (ACGME), [19] allowing for a unified set of competency areas (patient care, clinical knowledge, interpersonal and communication skills, professionalism, practice-based learning and improvement, systems-based practice) which could be assessed across disciplines.

Information about trainee and supervisor demographics and professional characteristics was also collected, including modality of supervision. Trainees were asked the question “Which of the following modalities of supervision have you and your VA supervising practitioner(s) used? [Check all that apply]” and supervisors were asked “Which of the following modalities of trainee supervision have you used? [Check all that apply].” Response options for both groups included: In-person supervision (meetings occur in person, face-to-face), Video virtual supervision (meetings occur virtually, using a method that allows video viewing of each other), and Telephone virtual supervision (meetings occur virtually, using a method that allows you to hear each other’s voices but not see any video).

A pilot study of VA trainees, supervisors, and one site educational officer was conducted at one high-complexity VA medical center in August 2022 to refine the survey instrument and the survey distribution plan, as well as identity potential issues with face validity before the full survey launch. In addition to taking the survey, pilot participants participated in feedback sessions to discuss confusing or ambiguous terms and questions, assess the flow and logic of the survey, and weigh in on the outreach plan. Feedback from the pilot informed decisions regarding language choice, such as not using the acronym “HPT” (health professions trainee) due to trainee unfamiliarity with the term. The pilot also informed the need to clarify in the survey introduction that the terms “attending” and “preceptor” can be referred to as a supervisor, the need to define the word “synchronous” in the supervision definition, and the openness of trainees and supervisors to an optional open-text box allowing respondents to share any additional feedback they may have regarding virtual supervision.

### Procedure

Survey administration was facilitated via direct outreach to site education leadership. Site leaders were asked to provide a list of pertinent program directors at their sites, who in turn were contacted to supply contact email addresses for the trainees and supervisors working within Primary Care and OPMH settings in the targeted disciplines. For trainees, this included VA and non-VA email addresses. The evaluation team then composed outreach emails for program directors to send to survey targets that included information about the evaluation, how respondent confidentiality would be protected, and an anonymous link to a Qualtrics web-based survey. For the trainee version of the survey, a QR code linking to the survey was also included in the outreach email. Surveys to both groups were distributed between January and May 2023. The evaluation team provided two reminder emails for program directors to send to trainees.

In addition to sending the trainee email survey, program directors were also asked to raise awareness of both the trainee and supervisor versions of the survey, dedicate 10 minutes of didactics sessions for trainees to take the survey, and provide a flyer containing the QR code linking to the survey in trainee common areas. These recommendations were intended to address barriers to survey completion and were offered based on feedback generated from supervisors participating in the one-site pilot administration of the survey instruments.

### Analysis

Given the low counts of those who had experienced exclusively virtual supervision, respondents were classified as either “in-person only,” indicating those who only selected “In-person supervision” when asked about their supervision modality, or “some virtual supervision,” which combined hybrid and virtual-only supervision. Independent samples t-tests were used to compare means and Cohen’s *d* measures of effect size were used to determine whether meaningful differences existed between the in-person only and some virtual groups. Individual rating items with numerical response formats of at least five categories in length were treated as continuous if the data appeared normally distributed [20]. Mean differences were considered statistically significant if *p*-values were less than a pre-specified alpha level of 0.01. Cohen’s *d* estimates were interpreted as follows: 0.2–0.5 was considered a “small effect,” 0.5–0.8 a “moderate effect,” and >0.8 a “large effect”. [21] Subgroup analyses by discipline were conducted, with mean differences and interaction *p*-values estimated for competency areas and other measures. All statistical analyses were conducted in Stata 18 MP (StataCorp).

Open-ended survey responses were analyzed using NVivo 12.0 software (Lumivero; formerly produced by QSR International) using inductive qualitative content analysis [22]. One coder (GY) first reviewed all open-ended responses, identifying and developing codes directly from the data. Two additional coders (KB, CAV) then reviewed the coding scheme to identify discrepancies and modify or expand the codebook when necessary. No discrepancies were found during this review.

### Ethical considerations

Per regulations outlined in VHA Program Guide 1200.21 [23], this evaluation was designated a non-research quality improvement activity. While the evaluation was exempt from IRB oversight, steps were taken to ensure the welfare of survey respondents. Trainees and supervisors were informed that participation was voluntary, responses were confidential, and survey results would be reported in aggregate. Survey content and methodology were reviewed and approved by the VA Organizational Assessment Committee (OAC) and VA Office of Labor Management Relations (LMR).

## Results

### Population characteristics

Among the 20 targeted sites, 17 sites supplied respondents. 13 sites had both trainees and supervisors respond, 3 sites had only trainees respond, and 1 site had only supervisors respond. In total, 181 trainees and 167 supervisors completed the survey. Respondent professional demographics are presented in Table 1. Among trainee respondents, psychology trainees provided a plurality of the responses (42.0%) followed by Internal Medicine trainees (31.5%). The largest proportion of trainees were in clinical training in VA for 6–12 months (47.8%) while 27.2% had been training in VA for more than 18 months, 15.0% less than 6 months, and 10.0% for 13–18 months. Among supervisor respondents, most reported being former VA trainees (72.3%). Psychology supervisors provided most responses (53.3%) followed by Psychiatry (19.2%). A plurality of supervisors reported training VA trainees for 10 or more years (32.3%). Among trainees, 35.4% had experienced exclusively in-person supervision, while 63.0% had experienced a hybrid of in-person and virtual supervision, operationalized as the use of telephone and/or video supervision. Only 1.7% of trainees had experienced exclusively virtual supervision. Among supervisors, 23.4% had exclusively experienced in-person supervision, 74.3% had experienced a hybrid of in-person and virtual supervision, and 2.4% had exclusively experienced virtual supervision. Among trainees, most respondents (64.6%) rated their supervision experience as including some virtual supervision during their training VA while 35.4% had only in-person supervision (Table 1). Most supervisors (76.7%) indicated some experience with virtual supervision in VA while 23.4% had conducted exclusively in-person supervision (Table 1).

**Table 1:** Professional characteristics of trainees and supervisors

	Trainees (*N* = 181)		Supervisors (*N* = 167)	
Characteristic	*N*	%	*N*	%
Discipline				
Internal Medicine	57	31.5%	26	15.6%
Family Medicine	21	11.6%	3	1.8%
Nursing	1	0.6%	5	3.0%
Psychiatry	19	10.5%	32	19.2%
Psychology	76	42.0%	89	53.3%
Multi-discipline	7	3.9%	4	2.4%
Other	0	0.0%	8	4.8%
Months as VA Health Professions Trainee				
Less than 6 months	27	15.0%	NA	NA
6–12 months	86	47.8%	NA	NA
13–18 months	18	10.0%	NA	NA
More than 18 months	49	27.2%	NA	NA
Previous VA Trainee				
Yes	NA	NA	120	72.3%
No	NA	NA	46	27.7%
Virtual Modality Experience				
In-person only	64	35.4%	39	23.4%
Some Virtual	117	64.6%	128	76.7%
Modality Breakdown				
In-person	178	98.3%	163	97.6%
Video	97	53.6%	115	68.9%
Phone	38	21.0%	50	29.9%
No. of Trainees Supervised in last year				
1	NA	NA	27	16.2%
2–4	NA	NA	70	41.9%
5–10	NA	NA	31	18.6%
11–15	NA	NA	10	6.0%
16–20	NA	NA	2	1.2%
20+	NA	NA	27	16.2%
Maximum no. of Trainees Supervised				
1	NA	NA	54	32.3%
2–4	NA	NA	92	55.1%
5–10	NA	NA	16	9.6%
11–15	NA	NA	5	3.0%
Proportion of VA Time to Supervise				
Less than 10%	NA	NA	78	47.0%
11–25%	NA	NA	56	33.7%
26–50%	NA	NA	19	11.5%
51–75%	NA	NA	9	5.4%
Greater than 75%	NA	NA	4	2.4%
Length of Time Supervising Trainees				
Less than 6 months	NA	NA	2	1.2%
6–12 months	NA	NA	11	6.6%
1–2 years	NA	NA	17	10.2%
3–5 years	NA	NA	35	21.0%
6–10 years	NA	NA	48	28.7%
More than 10 years	NA	NA	54	32.3%

Hybrid and virtual supervision were combined for analysis due to the low number of respondents who only had experience with virtual supervision (*N* = 4 virtual-only trainees; *N* = 4 virtual-only supervisors)

### Equivalence tests comparing some virtual supervision to in-person only supervision

#### Competency development

Analyses focused on assessing potential differences in perceived competency levels of trainees on ACGME competency skill areas based on ratings from trainees who received some virtual supervision (“some virtual,” *N* = 117) and only in-person supervision (“in-person”, *N* = 64). Competency rating scales ranged from 0 to 10, with higher scores reflecting greater perceived competency in that clinical area. Levels of perceived competency development were highly rated by both some virtual trainees (average competency ratings: 8.39–8.77 across areas) and in-person only trainees (average competency ratings: 7.61–7.94 across areas), although some virtual trainees had significantly higher than average competency ratings than in-person only trainees for patient care, knowledge for practice, interpersonal and communication skills, practice-based learning and improvement, and systems-based practice (Table 2). The groups did not significantly differ in terms of perceived competency in professionalism.

**Table 2:** Perceived ratings of trainee clinical competency and preparedness to respond to patient emergencies among trainees and supervisors, stratified by supervision type

Ratings of Clinical Competency					
Trainees (*N* = 181)	Hybrid/Virtual	In-person			
Competency area^1^	Mean (SD)	Mean (SD)	Difference (95% CI)	t	Effect Size^2^
Patient care	8.63 (1.62)	7.78 (1.96)	0.85 (0.32, 1.39)	3.14^**^	0.49
Knowledge for practice	8.77 (1.42)	7.78 (1.79)	0.99 (0.51, 1.47)	4.07^**^	0.63
Interpersonal and communication skills	8.39 (1.65)	7.61 (2.02)	0.78 (0.23, 1.33)	2.82^**^	0.44
Professionalism	8.56 (1.77)	7.94 (1.87)	0.62 (0.06, 1.17)	2.20	0.34
Practice-based learning and improvement	8.69 (1.45)	7.80 (1.88)	0.90 (0.40, 1.39)	3.56^**^	0.55
Systems-based practice	8.68 (1.47)	7.59 (2.17)	1.08 (0.55, 1.62)	3.99^**^	0.62

Supervisors (*N* = 167)	**Hybrid/Virtual**	**In-person**			
**Competency area** ^**1**^	**Mean (SD)**	**Mean (SD)**	**Difference (95% CI)**	***t***	**Effect Size** ^**2**^
Patient care	8.31 (1.03)	8.00 (0.97)	0.31 (−0.06, 0.68)	1.68^**^	0.31
Knowledge for practice	8.05 (1.12)	7.51 (1.10)	0.53 (0.13, 0.94)	2.62	0.48
Interpersonal and communication skills	8.34 (1.15)	8.10 (1.19)	0.23 (−0.18, 0.65)	1.10	0.20
Professionalism	8.48 (1.32)	8.10 (1.43)	0.37 (−0.11, 0.86)	1.52	0.28
Practice-based learning and improvement	8.27 (1.22)	7.64 (1.16)	0.62 (0.19, 1.06)	2.83^**^	0.52
Systems-based practice	7.77 (1.41)	7.03 (1.27)	0.74 (0.24, 1.24)	2.94^**^	0.54

**Ratings of Preparedness to Respond to Patient Emergencies**					
Trainees (*N* = 181)	**Hybrid/Virtual**	**In-person**			
	**Mean (SD)**	**Mean (SD)**	**Difference (95% CI)**	***t***	**Effect Size** ^**2**^
Readiness to respond to patient emergencies^3^	4.05 (0.92)	3.52 (1.01)	0.54 (0.27, 0.81)	3.90^**^	0.61

Supervisors (*N* = 167)	**Hybrid/Virtual**	**In-person**			
	**Mean (SD)**	**Mean (SD)**	**Difference (95% CI)**	***t***	**Effect Size** ^**2**^
Readiness to respond to patient emergencies^3^	4.34 (0.64)	3.80 (0.80)	0.54 (0.29, 0.79)	4.33^**^	0.79

^1^Competency ratings based on self-reported endorsement of items on a 0–10 scale. ^2^Effect size strength: 0.2–0.5: “small effect,” 0.5–0.8: “moderate effect,” 0.8–1.0: “large effect.” ^3^Readiness to respond to patient emergencies rated on a 1–5 scale

**p <.01

Supervisors responded with similarly high levels of perceived clinical competency development by their trainees for both some virtual supervisors (average competency ratings: 7.77–8.48 across areas) and in-person only (average competency ratings: 7.03–8.10 across areas). Supervisors with some virtual supervision rated their trainees as having significantly higher levels of competency development in terms of patient care, practice-based learning and improvement, and systems-based practice, but were equivalent in terms of level of perceived competence related to knowledge for practice, interpersonal and communication skills, and professionalism (Table 2).

#### Preparedness for patient emergencies

Trainees and supervisors also provided feedback on their perceptions of the extent to which their VA supervision experience allowed them to develop skills to effectively respond to patient emergencies. Both trainee and supervisor ratings were provided on a scale with score options from 1 to 5, with higher scores reflecting a greater level of perceived preparedness to respond to patient emergencies. Trainees who experienced some virtual supervision rated themselves as, on average, significantly more prepared to respond to patient emergencies than did in-person only trainees, and supervisors with some virtual supervision similarly rated their trainees as significantly more prepared to respond to an emergency than did in-person only supervisors (Table 2).

#### Supervisory working relationships

Additional analyses focused on trainee and supervisor perceptions of their working relationships. Trainee and supervisor ratings were provided on a scale with score options from 1 to 5, with higher scores reflecting a greater level of agreement with that aspect of the supervisory relationship. Both trainees and supervisors provided high average ratings for all aspects of the supervisory relationship, although some virtual trainees had higher average ratings related to (1) collaborative decision-making, (2) the perception that their supervisor had their best interests in mind, (3) satisfaction with their VA supervision, (4) the perception that they receive useful feedback about their VA performance, (4) opportunities discuss topics related to professional development, and (5) satisfaction with frequency of supervision meetings (Table 3). In-person only and some virtual trainees were equivalent in terms of their average ratings of other aspects concerning supervisory working relationships, and supervisors were equivalent in their average ratings of all aspects of supervisory working relationships.

**Table 3:** Ratings of quality of working relationship between supervisor and Trainee, stratified by modality of supervision

Trainees (*N* = 181)	Hybrid/Virtual	In-person			
Relationship characteristic^1^	Mean (SD)	Mean (SD)	Difference (95% CI)	t	Effect Size^2^
My VA supervising practitioner(s) and I effectively collaborate to support patient care decisions.	4.83 (0.44)	4.45 (0.85)	0.37 (0.18, 0.56)	3.88^**^	0.60
I feel that my VA supervising practitioner(s) have my best interests in mind.	4.71 (0.63)	4.41 (0.85)	0.30 (0.08, 0.52)	2.69^**^	0.42
I feel respected and heard by my VA supervising practitioner(s).	4.66 (0.69)	4.38 (0.88)	0.29 (0.05, 0.52)	2.41	0.38
I feel comfortable working with my VA supervising practitioner(s).	4.67 (0.68)	4.58(0.77)	0.09 (−0.13, 0.31)	0.85	0.13
My VA supervising practitioner(s) trust my decisions related to patient care	4.68 (0.61)	4.45 (0.78)	0.23 (0.02, 0.44)	2.17	0.34
Overall, I am satisfied with my supervision at VA.	4.62 (0.73)	4.28 (0.98)	0.34 (0.09, 0.60)	2.67^**^	0.41
My VA supervising practitioner(s) have an accurate view of my performance.	4.56 (0.75)	4.25 (0.89)	0.31 (0.06, 0.55)	2.45	0.38
I receive useful feedback about my performance within my VA supervision.	4.59 (0.72)	4.03 (1.14)	0.56 (0.29, 0.83)	4.03^**^	0.63
My VA supervision allows for opportunities for professional modelling by my supervising practitioner(s).	4.50 (0.75)	4.2 (0.91)	0.29 (0.04, 0.54)	2.32	0.36
My VA supervision allows for opportunities to discuss topics related to professional development.	4.57 (0.73)	4.19 (0.91)	0.39 (−0.14, 0.63)	3.10^**^	0.48
I am satisfied with the frequency that I meet with my VA supervising practitioner(s).	4.64 (0.71)	4.33 (0.84)	0.31 (0.08, 0.55)	2.65^**^	0.41
I am satisfied with the amount of time I spend with my VA supervising practitioner(s).	4.62 (0.73)	4.36 (0.84)	0.26 (0.03, 0.05)	2.21	0.34

Supervisors (*N* = 167)	**Hybrid/Virtual**	**In-person**			
**Relationship characteristic** ^**1**^	**Mean (SD)**	**Mean (SD)**	**Difference (95% CI)**	***t***	**Effect Size** ^**2**^
I effectively collaborate with my trainees to support patient care decisions.	4.91 (0.32)	4.87 (0.34)	0.03 (−0.08, 0.15)	0.58	0.11
The trainees that I supervise feel I have their best interests in mind.	4.82 (0.41)	4.77 (0.48)	0.05 (−0.10, 0.20)	0.66	0.12
The trainees that I supervise feel respected and heard.	4.86 (0.35)	4.77 (0.43)	0.09 (−0.04, 0.22)	1.34	0.24
I feel comfortable working with my trainees.	4.89 (0.31)	4.85 (0.37)	0.04 (−0.07, 0.16)	0.75	0.14
I trust my trainees’ decisions related to patient care.	4.58 (0.57)	4.46 (0.60	0.12 (−0.09, 0.32)	1.10	0.20
Overall, I am satisfied with my supervisory activities at VA.	4.73 (0.51)	4.54 (0.68)	0.19 (−0.01, 0.39)	1.85	0.34
I am able to effectively get an accurate view of my trainees’ performance during supervision sessions.	4.67 (0.49)	4.62 (0.54)	0.06 (−0.12, 0.24)	0.62	0.11
I am able to effectively provide useful performance feedback to my trainees during supervision sessions.	4.70 (0.52)	4.56 (0.64)	0.14 (−0.06, 0.34)	1.38	0.25
I am able to effectively model professional standards to my trainees during supervision sessions.	4.80 (0.48)	4.69 (0.52)	0.10 (−0.07, 0.28)	1.18	0.22
I am able to effectively discuss topics related to professional development with my trainees during supervision sessions.	4.70 (0.62)	4.56 (0.64)	0.13 (−0.09, 0.36)	1.15	0.21
I am satisfied with the frequency that I meet with my trainees.	4.78 (0.47)	4.64 (0.63)	0.14 (−0.04, 0.32)	1.50	0.28
I am satisfied with the amount of time I spend with my trainees.	4.73 (0.50)	4.56 (0.68)	0.16 (−0.03, 0.36)	1.63	0.30

^1^Supervisor/trainee relationship qualities rated on a 1–5 scale. ^2^Effect size strength: 0.2–0.5: “small effect,” 0.5–0.8: “moderate effect,” 0.8–1.0: “large effect”

**p <.01

#### Subgroup differences

Additional sensitivity analyses were conducted to investigate whether a preference for virtual versus in-person supervision varied across disciplines. While no differences were found among supervisors in terms of trainee skill development or supervisory relationship quality based on discipline or among trainees in terms of skill development, psychology trainees were found to have a significantly higher preference for virtual training versus in-person training relative to other disciplines for the following relationship areas: effective collaboration in patient care decisions (rating difference, some virtual versus in-person only: Internal Medicine: MDif = 0.17; Psychology: MDif = 1.51; Other, MDif = 0.13, interaction *p*-value = 0.000), comfort in working with supervisor (rating difference, some virtual versus in-person only: Internal Medicine: MDif = 0.04; Psychology: MDif = 0.96; Other, MDif = −0.31, interaction *p*-value = 0.005), and satisfaction with amount of time spent with supervisor (rating difference, some virtual versus in-person only: Internal Medicine: MDif = −0.10); Psychology: MDif = 1.18; Other, MDif = 0.07, interaction *p*-value = 0.006).

### VA trainee and supervisor perspectives related to the use of virtual supervision

A select number of questions on both the trainee and supervisor surveys were asked only to respondents who had experience with virtual supervision (*N* = 117 trainees; *N* = 128 supervisors). These questions elicited perspectives on how virtual supervision contributes to VA training and challenges related to the use of virtual supervision.

#### Impacts of virtual supervision

Most trainees and supervisors who had virtual supervision experience did not feel it interfered with their VA training; 65.8% of trainees and 62.5% of supervisors endorsed the response “not at all” when asked about the extent to which virtual supervision negatively interferes with VA training. Conversely, most trainees and supervisors felt that virtual supervision contributed to their VA training; 69.2% of trainees and 84.4% of supervisors endorsed the responses “a lot” or “completely” when asked the extent to which virtual supervision positively contributes to their training.

#### Challenges related to virtual supervision

In addition to being asked about how virtual supervision affects VA training, trainees and supervisors who had some virtual supervision were asked about potential challenges encountered during virtual supervision. Common challenges endorsed by trainees included internet connectivity and bandwidth issues (40.2%), difficulties with software applications (38.5%), limited access to VA’s network (22.2%), hardware issues (18.8%), challenges in finding private space for remote supervision (17.1%), and non-work environmental disruptions (16.2%). Approximately 30% (29.9%) of trainees indicated that none of these issues presented challenges to remote supervision sessions. For supervisors, the most endorsed challenge was internet connectivity and bandwidth issues (31.3%), followed by challenges of software programs (28.9%), sessions interrupted by non-work environmental distractions (17.7%), lack of reliable access to VA’s network (11.7%), hardware issues (10.9%), and inability to find a private space for remote supervision (7.8%). Notably, a large proportion of supervisors indicated that none of these factors presented challenges during their virtual supervision sessions (49.2%) (Table 4).

**Table 4:** Challenges encountered during virtual Supervision, stratified by role (Trainees vs. Preceptors)

Disruption	Trainees	Preceptors	Chi-square
Internet connectivity/bandwidth issues	47 (40.2)	40 (31.3)	2.12
Non-work environmental distractions	19 (16.2)	22 (17.2)	0.04
Software applications freezing, locking, not launching, etc.	45 (38.5)	37 (28.9)	2.51
Hardware issues	22 (18.8)	14 (10.9)	3.02
Inability to find private space for remote supervision	20 (17.1)	10 (7.8)	4.9
Lack of reliable access to VA networks	26 (22.2)	15 (11.7)	4.84
Other	0 (0.0)	5 (3.9)	4.67
None of the above	35 (29.9)	63 (49.2)	9.50**

**p <.01

#### Qualitative findings

Perspectives related to the use of virtual supervision were also assessed qualitatively. 19.9% of trainee respondents (*n* = 36) and 51.4% of supervisor respondents (*n* = 86) provided feedback to the question “*What feedback do you have to OAA about the role of virtual supervision in VA*?”. Among trainees, four primary domains were identified in the content analysis: (1) benefits of virtual supervision, (2) positive experience with virtual supervision, (3) negative impact of virtual supervision, and (4) continuation of virtual supervision. Among supervisors, four primary domains were identified in the content analysis: (1) benefits of virtual supervision, (2) negative impact of virtual supervision, (3) consequences of removing virtual supervision, and (4) recommendations.

Overall, trainees found virtual supervision to be a very flexible and convenient supervisory modality, with several trainees noting that it enhances accessibility to their supervisors. Further, trainees reported that virtual supervision provides easier access to supervisor support, is more convenient for quick or emergency questions, and saves time on trying to find office space to meet in-person. Trainees also noted that virtual supervision decreases travel time between sites, is beneficial for last minute scheduling changes, and is more convenient when facing back-to-back meetings. However, others reported concerns that virtual supervision could impede on mentorship and social support during their training experience. Interestingly, and in contrast to most responses, one trainee noted that they did not think that virtual supervision “played a role at all” in their VA training. Despite positive reports on virtual supervision, comments were mixed on preferences between virtual supervision and in-person supervision. Trainees did not provide any recommendations for improvements to virtual supervision moving forward.

Supervisors identified both benefits and concerns associated with virtual supervision. This group reported that virtual supervision increases trainee accessibility to supervisors and provides greater flexibility for training trainees. Notably, they reported that virtual supervision expands training opportunities, particularly in terms of resource sharing, problem-solving, screen sharing for chart reviews, and observations of patient encounters. It was further noted that virtual supervision provides greater flexibility in scheduling supervisory meetings, as it allows for a wider range of meeting times and eliminates the need to find private physical space to meet, while being beneficial for recruiting and retaining supervisory practitioners. Despite the benefits associated with virtual supervision, supervisors also reported some concerns, notably that it presents additional technological issues and may impede on ability to assess trainee behavior. Interestingly, many supervisors said that they preferred in-person supervision to virtual supervision; however, many also reported that removing virtual supervision as an option would increase burden on staff and would make the VA less competitive with other training programs. Moving forward, supervisors had several recommendations for improving the virtual supervision experience for both supervisors and trainees, including fixing major technological issues, providing training to virtual supervisors on best practices, and recognizing that supervision needs may vary across disciplines, with psychology called out as one discipline in which virtual supervision can be especially effective. More details on these findings can be found in Table 5.

**Table 5:** Identified trainee and supervisor domains

Domain	Key Concepts	Exemplar Quotes
*Identified Trainee Domains*		
Benefits of virtual supervision	• Flexible • Convenient • Safe • Increased supervisor availability	*“I appreciate the flexibility that is provided with virtual supervision*,* such [as] decreasing travel time between sites*,* consulting outside of scheduled supervision times*,* last minute changes in scheduling with patients and other responsibilities*,* and when having back to back meetings.”* *“I have been able to access my supervisors more frequently and readily with virtual supervision.”*
Positive experience with virtual supervision	• Fitting for VA setting • Good alternative to in-person	*“Virtual supervision is an asset. I have found it extremely beneficial*,* progressive*,* and fitting for the VA setting. I hope that this modality of supervision continues.”* *“It [virtual supervision] is vital for evolving practice requirements.”*
Negative impact of virtual supervision	• Lack of mentorship • Lack of social support	*“I think in general virtual work should be minimized in the training setting because it does not allow for the same level of mentorship and social support during this intense and demanding experience.”*
Continuation of virtual supervision	• Support continued use	*“I think virtual supervision could be implemented more frequently into the norm of supervision.”* *“Virtual supervision is a helpful resource to have available. It allows for flexibility and easy access to support. Given the demands placed on trainees and mental health staff*,* having virtual platforms as an option is extremely helpful and useful.”*
*Identified Supervisor Domains*		
Benefits of virtual supervision	• Expands training opportunities • Expands supervision participation • Flexible • Accessible • Helpful to recruitment and retention efforts	*“I think virtual supervision makes supervisors more accessible and gives the faculty and trainees a level of flexibility that they desire without having a negative impact on patient care.”* *“I think virtual supervision is critical to maintaining our training program. At this point*,* given that many practitioners telework 50% of their time*,* it is unrealistic/unpractical to have no supervision virtually and that many providers will choose not to supervise as a result.”*
Negative impact of virtual supervision	• Lack of hands-on learning • Unable to assess HPT behavior • Technology issues	*“There is something lost from not being able to see the whole trainee and their body language during meetings and/or note if they are multitasking.”* *“I know the world is changing and we are moving towards virtual but there is a role for being present wit the trainee and caring for the patient together. As we care for the patient together the trainee observes and is able to learn from the supervising attending. It is a seamless process that cannot be separated into blocks of time. Supervision is not mutually exclusive.”*
Consequences of removing virtual supervision	• Increases burden • Reduces training opportunities • Less supervisor availability • VA will be less competitive	*“If we no longer had virtual supervision flexibilities at VA I think it would do great harm to our training programs. It places more burden on both supervisors and staff*,* and I’m certain that other settings will be continuing with virtual supervision. Thus*,* if we lose this functionality*,* we will not only be making VA less competitive with other training sites/programs*,* but we will also be missing an opportunity to prepare our trainees for the new occupational reality.”* *“Increasingly*,* VA is hiring health professionals that work full- or part-time virtually. If VA doesn’t adopt the use of virtual supervision*,* then those supervisors will not be part of training. This reduces the training opportunities for trainees and increases supervision demands on those staff who do not work virtually.”*
Recommendations	• Fix technology issues • Allow programs to decide which modality to use • Provide more training to supervisors on virtual supervision • Set same expectations for both supervisors and trainees	*“If virtual supervision is authorized*,* I would recommend a mandated virtual supervisory training since it does require additional skills from in-person supervision.”* *“There should be recognition that supervision needs may differ across disciplines*,* especially between trainees who do work involving physical exam vs. those that do not.”* *“Virtual supervision is vital for psychology. It allows for flexibility and better trainee satisfaction.”*

## Discussion

### Comparisons to in-person supervision

This evaluation found strong support for virtual supervision among VA trainees, corroborating existing scholarship showing high support for virtual supervision among VA trainees [6]. Also in line with previous literature, [10, 13, 14] virtual supervision was found as effective as in-person supervision at establishing effective working relationships. Some amount of virtual supervision may be even *more* effective at establishing effective supervisory relationships between trainees and supervisors than exclusively in-person supervision.

This evaluation also found that virtual supervision was an effective way to support the development of trainee clinical competencies across a variety of skill areas from both the perspective of trainees and supervisors. Trainees who received virtual supervision achieved equivalent or more advanced levels of skill development than those who received in-person supervision alone. It is especially notable that both trainees and supervisors rated those who received virtual supervision as achieving a higher level of skill development in terms of patient care (presumably, the most important single goal area for the development of clinical trainees) and ability to respond to patient emergencies (which may require the most advanced and flexible clinical skillset) than trainees who received in-person supervision alone. This finding highlights the potential contributions of virtual supervision in the development of essential clinical skills. To the authors’ awareness, these represent novel findings regarding the use of virtual training to support clinical skill development. These findings can inform medical education curricula going forward, as trainees and supervisors may find virtual supervision more convenient and effective than in-person supervision for certain settings.

Notably, neither “in-person only” trainees nor “in-person only” supervisors had significantly higher ratings on outcome measures related to competency development, preparation to address patient emergencies, and supervisory working relationships than their “some virtual supervision” counterparts, suggesting that for the populations surveyed, the two groups were equivalent on these outcomes. This pattern of findings could point to the value of increased opportunities for independence within a virtual supervision environment, which may allow trainees to utilize clinical judgement, deliver interventions, and refine clinical skills more extensively than is possible in a traditional in-person training environment which includes more formal oversight of clinical activities (and more active involvement by supervisors). Continuing this line of thought, it is notable that virtual trainees were rated as having higher levels of skill areas related to care delivery, emergency response, and skill application, but not as being more professional than exclusively in-person trainees. Increased clinical independence may also lead trainees to have greater confidence in their own abilities and higher levels of collaboration effectiveness with their supervisors, as reflected by their higher self-rated competencies and higher ratings of supervisory relationship aspects related to clinical collaboration relative to in-person trainees. However, it may also be that more advanced and skilled trainees are more likely to be offered virtual supervision opportunities than less skilled trainees, which may also contribute to the pattern of results. A lack of significant difference in supervisor ratings of the quality of the supervisory relationship between virtual and in-person trainees is likely due to ceiling effects and positive global perspectives of supervisory relationships, as reflected by average scores near the maximum possible value for all aspects of the supervisory relationship.

Comparisons looking at the intersection of clinical discipline and differences between ratings of perceived trainee clinical skill development and the quality of supervisory relationships between some virtual supervisors and in-person only supervisors showed consistent differences across disciplines for supervisors, suggesting that the results presented previously are representative of supervisors across discipline. Similarly, some virtual trainees and in-person only trainees provided similar differences with regard to clinical skill competence, suggesting similar perspectives across disciplines. However, psychology trainees provided ratings of several aspects of their supervisory relationships (effective collaboration in patient care decision, comfort in working with supervisor, satisfaction with amount of time spent with supervisor) which were significantly larger for trainees with some virtual supervision relative to in-person only trainees, when compared to trainees of other disciplines. This finding suggests that the use of virtual supervision was viewed as especially impactful in supporting the development of effective supervisory relationships for psychology trainees in this study.

### Challenges and benefits of virtual supervision

Some challenges to virtual training were noted, internet connectivity and bandwidth issues, difficulties with software applications, and limited access to VA’s network. However, most trainees and supervisors who had participated in virtual supervision held a positive view toward this modality of instruction. Majorities in both groups felt that virtual supervision made important contributions to VA training, while separately noting that virtual supervision did not interfere with other VA training activities. In line with a previous study, [24] benefits of virtual supervision in the VA context included flexibility and convenience, expanded training opportunities, and increased access to supervisors, as well as challenges such as technological failures and limitations to the supervisory relationship, such as a perceived lack of mentorship and support.

### Limitations

Like other studies, this evaluation had limitations. There may be unmeasured factors which may have contributed to the decision of whether trainees were good candidates for virtual supervision which may have also been related to our outcomes of interest, such as prior clinical training, maturity, or pre-training skill development. It is also possible that trainees and supervisors who chose to participate in the evaluation may be misrepresentative of the larger VA training community in ways that could impact their participation in virtual supervision or their view of clinical skill development. Further, while the evaluation team intended to gather trainee and supervisor responses across all sampled sites, three sites that agreed to participate in the evaluation did not produce any trainee or supervisor responses. Total trainee and supervisor response rates also could not be calculated; only 12 sites submitted counts for the number of eligible trainees during the evaluation period of interest and only 9 sites submitted counts for the number of eligible supervisors. The evaluation was further limited in that trainee and supervisor data were collected separately and could not be analyzed as supervisory dyads. Sample sizes were also small compared to the number of trainees in the VA system. As such, caution is warranted in generalizing findings to the broader population of VA trainees and supervisors.

### Recommendations for additional study and training guidance

This evaluation resulted in several recommendations for additional study of virtual supervision and more direct guidance on the use of virtual supervision. Firstly, it is recommended that other studies expand on the potential equivalence of in-person and virtual supervision using other qualitative methods (e.g., focus group sessions, semi-structured interviews), which may identify other important factors which were not identified in this evaluation. Such efforts may be particularly helpful given the low response rate achieved by the present evaluation. Additionally, as the current evaluation focused on a limited number of VA trainees and supervisors, future evaluations of this topic would benefit from an expanded scope which considers the value of virtual supervision for other clinical supervisor and trainee groups (e.g., Social Work). It may also be useful to consider the perspectives of other key partners such as non-VA university affiliates and licensing organizations related to the use of virtual supervision and any concerns that these groups may have related the use of virtual supervision within VA training programs.

Notably, this evaluation identified a connection between virtual supervision and the ability to recruit and retain VA supervisors that, to the authors’ knowledge, has not been previously discussed extant literature. Future studies could build upon this finding through further investigation of the impact of virtual supervision on healthcare recruitment and retention, as well as whether the benefits of virtual supervision extend beyond mention of workplace flexibility to reduced clinician burnout and improved well-being.

Additionally, evaluation related to a minimum amount of in-person contact between trainee and supervisor could help trainees and supervisors experience the benefits of both in-person and virtual training collaborations, by perhaps focusing on the most technical aspects of the training placement. Considering the feedback from some respondents that virtual supervision may result in a lack of hands-on training opportunities or limited mentorship opportunities, it may be helpful for healthcare educators to advocate for hybrid rather than fully virtual supervision arrangements in many trainee placements. As the ideal balance between in-person and virtual supervision will likely vary considerably across disciplines, it would be value-added to include multiple disciplines in future studies aimed at making broad recommendations to the field.

Future studies could also further explore the current evaluation’s finding that psychology trainees had a significantly higher preference for virtual training versus in-person training relative to other disciplines in select relationship areas, looking at whether this finding can be replicated and what the underlying reasons for this difference may be.

In addition to recommendations related to further study, findings from this evaluation merit additional discussion offices overseeing training programs. For example, given the high rates of endorsed barriers to virtual supervision participation endorsed by trainees and supervisions, it is recommended that offices overseeing training programs provide guidance related to supervisor and trainee expectations/requirements for participation in virtual supervision. Many of the factors indicated by respondents as impacting their virtual supervision experience (e.g., inability to find a private space for virtual supervision) could likely be avoided or minimized if there was a structured process in place that trainees and supervisors needed to complete before initiating virtual supervision. Such a “set up” process could also assist supervisors and technical support persons in troubleshooting for other barriers to virtual supervision before they emerge, such as challenges with hardware or software.

Lastly, given the qualitative feedback presented here, supervisors may also benefit from additional training and preparation related to the assessment of trainee behavior. At present, training of VA supervisors regarding how to conduct and assess virtual supervision, as well as any preparation VA trainees have before they receive virtual supervision, is handled at the local site or disciplinary department level.

## Conclusions

This evaluation adds to the body of knowledge on the equivalence of virtual and in-person supervision, constituting the first evaluation of the equivalency of experiences of trainees and supervisors who engaged in some virtual supervision in terms of clinical competency development, ability to respond to patient emergencies, and the establishment of effective trainee/supervisor working relationships, as well as perspectives on virtual supervision from VA trainees in multiple disciplines. Some virtual supervision was equivalent or better than exclusive in-person supervision in supporting clinical skill development and the establishment of effective supervisory relationships. While further evaluation is needed to examine the impacts of virtual supervision on trainees beyond subjective assessment, our evaluation suggests that some level of virtual supervision can be incorporated as a standard aspect of health professions training programs without affecting important areas of trainee clinical and professional development.

## Supplementary Information

Supplementary Material 1.

Supplementary Material 2.

## Data Availability

The datasets generated and/or analyzed during the current study are not publicly available. Data are however available from the authors upon reasonable request and with permission of the VA QUERI Center for Evaluation and Implementation Resources (CEIR) and the VA Office of Academic Affiliations (OAA).

## References

[1] Phillips LA, Logan JN, Mather DB. COVID-19 and beyond: telesupervision training within the supervision competency. Train Educ Prof Psychol. 2021;15(4):284–9. 10.1037/tep0000362.

[2] Watters Y, Northey WF Jr. Online telesupervision: competence forged in a pandemic. J Fam Psychother. 2020;31(3–4):157–77. 10.1080/08975353.2020.1818500.

[3] Augusterfer EF, O’Neal CR, Martin SW, Sheikh TL, Mollica RF. The role of telemental health, tele-consultation, and tele-supervision in post-disaster and low-resource settings. Curr Psychiatry Rep. 2020;22(12):85. 10.1007/s11920-020-01209-5. 33247315 10.1007/s11920-020-01209-5 PMC7695585

[4] Bernhard PA, Camins JS. Supervision from afar: trainees’ perspectives on telesupervision. Couns Psychol Q. 2021;34(3–4):377–86. 10.1080/09515070.2020.1770697.

[5] Chipchase L, Hill A, Dunwoodie R, et al. Evaluating telesupervision as a support for clinical learning: an action research project. International Journal of Practice-based Learning in Health and Social Care. 2014;2(2):40–53.

[6] Harada N, Falco K, Bowman M, Byrne J. Telehealth and Virtual Supervision Practices for Health Professions Education in the Department of Veterans Affairs. *Available Res Sq Httpsdoiorg1021203rs3rs-4882752v1*. 26 September 2024, PREPRINT (Version 1). 10.1186/s12909-025-06698-7 PMC11863835 40011902

[7] Perle JG, Zheng W. A primer for Understanding and utilizing telesupervision with healthcare trainees. J Technol Behav Sci Published Online May 19, 2023:1–7. 10.1007/s41347-023-00322-5. 10.1007/s41347-023-00322-5 PMC10196304 37362064

[8] Wood JAV, Miller TW, Hargrove DS. Clinical supervision in rural settings: a telehealth model. Prof Psychol Res Pract. 2005;36(2):173–9. 10.1037/0735-7028.36.2.173.

[9] Inman AG, Soheilian SS, Luu LP, Telesupervision. Building bridges in a digital era. J Clin Psychol. 2019;75(2):292–301. 10.1002/jclp.22722. 30589439 10.1002/jclp.22722

[10] Jordan SE, Shearer EM. An exploration of supervision delivered via clinical video telehealth (CVT). Train Educ Prof Psychol. 2019;13(4):323–30. 10.1037/tep0000245.

[11] Schmittel EM, Lettenberger-Klein C, Oliver T, Butterfras RF, Adamson DW. Intentionality in academic telesupervision: a phenomenological study of faculty telesupervisors’ experiences. Contemp Fam Ther. 2023;45(1):61–74. 10.1007/s10591-021-09601-w. 34393358 10.1007/s10591-021-09601-w PMC8346778

[12] Soheilian SS, O’Shaughnessy T, Lehmann JS, Rivero M. Examining the impact of COVID-19 on supervisees’ experiences of clinical supervision. Train Educ Prof Psychol. 2023;17(2):167–75. 10.1037/tep0000418.

[13] Tarlow KR, McCord CE, Nelon JL, Bernhard PA. Comparing in-person supervision and telesupervision: a multiple baseline single-case study. J Psychother Integr. 2020;30(2):383–93. 10.1037/int0000210.

[14] Thompson SM, Keenan-Miller D, Dunn D, et al. Preferences for and acceptability of telesupervision among health service psychology trainees. Train Educ Prof Psychol. 2023;17(3):221–30. 10.1037/tep0000415.

[15] Tarlow KR, McCord CE, Nelon JL, Bernhard PA. Comparing in-person supervision and telesupervision: A multiple baseline single-case study. J Psychother Integr. 2020;30(2):383-393. 10.1037/int0000210

[16] Bowersox NW, Jedele JM, Kawentel LM. Quality enhancement research initiative (QUERI) program evaluation guide: developing an implementing a High-Quality evaluation. Administration Office of Research and Development; 2021. https://www.queri.research.va.gov/tools/QUERI-Evaluation-Guide.pdf. United States Department of Veterans Affairs Health.

[17] Additional OPES, Resources. Facility Complexity Model and Patient-level Risk Adjustment Models. VHA OPES. Accessed August 1, 2022. https://dvagov.sharepoint.com/sites/VHAOPES/SitePages/Facility-Complexity-Model-and-Patient-Level-Risk-Adjustment-Models.aspx

[18] Miech EJ, Freitag MB, Evans RR, et al. Facility-level conditions leading to higher reach: a configurational analysis of National VA weight management programming. BMC Health Serv Res. 2021;21(1):1–797. 10.1186/s12913-021-06774-w. 34380495 10.1186/s12913-021-06774-w PMC8359110

[19] Kavic MS. Competency and the six core competencies. JSLS. 2002;6(2):95–7. 12113429 PMC3043418

[20] Harpe SE. How to analyze likert and other rating scale data. Curr Pharm Teach Learn. 2015;7(6):836–50. 10.1016/j.cptl.2015.08.001.

[21] Cohen J. *Statistical Power Analysis for the Behavioral Sciences*. 1st edition. Academic Press; 1969. 10.4324/9780203771587.

[22] Elo S, Kyngäs H. The qualitative content analysis process. J Adv Nurs. 2008;62(1):107–15. 10.1111/j.1365-2648.2007.04569.x. 18352969 10.1111/j.1365-2648.2007.04569.x

[23] Affairs VHAOA, Office of Research and Development. 2019:13. https://www.research.va.gov/resources/policies/ProgramGuide-1200-21-VHA-Operations-Activities.pdf

[24] Shearer EM, Jordan SE, Eliason KD, et al. Perspectives of psychology supervisors and trainees: implications for supervision and telesupervision. J Technol Behav Sci. 2024;9(1):68–82. 10.1007/s41347-024-00387-w.

