# Supplementary material for: An evaluation of virtual supervision effectiveness within department of veterans affairs (VA) health professionals training programs
Source: BMC Med Educ. 2025 Oct 24;25:1490. doi: 10.1186/s12909-025-08062-1 (PMC12551159; doi:10.1186/s12909-025-08062-1)
Supplement: Supplementary file 1 — Supplementary Material 1. [file 12909_2025_8062_MOESM1_ESM.docx]

Supplemental File 1. OAA Virtual Supervision Survey – Trainee Version

Start of Block: Block 1 - Introduction

This survey is part of an evaluation assessing the impact of supervision modality (e.g., in-person supervision, video supervision, telephone supervision) on the professional development and skill acquisition of VA health professions trainees (including residents, interns, fellows, and students). This survey is being conducted by the VA QUERI Center for Evaluation and Implementation Resources (CEIR) in partnership with VA's Office of Academic Affiliations (OAA). Data from the survey will be used to help improve VA's educational programs for trainees. As someone who has been a trainee within the VA system, your perspective is important to a successful evaluation. Participation in the survey is completely voluntary. If you choose to participate, you may stop at any point if you no longer wish to continue. However, we do hope that you will decide to complete the survey in full before the close date of March 17, 2023. Your responses to the survey are also completely confidential. Only CEIR staff working on this evaluation will have access to individual survey responses. All results from this survey will be reported in aggregate.  The survey should take approximately 10 minutes. Should you have any questions about this survey, your participation, or the evaluation itself, please contact CEIR at ceirfeedback@va.gov. Thank you for your participation! Please click the NEXT button at the bottom right to begin the survey and advance through the questions.

End of Block: Block 1 - Introduction

Start of Block: Block 2 - Supervision definitions

definitions Please keep the following definition in mind as you complete the survey:     **Supervising practitioner**: A supervising practitioner is a VA staff provider who is credentialed and granted authority to provide care for patients by the VA medical facility and is granted authority to supervise trainees by the health professions education program in accordance with accrediting body standards. (E.g., attendings, preceptors).  **Supervision**: Supervision is defined as interaction between a trainee and supervising practitioner during or adjacent to an episode of care (e.g., procedures or encounters). Supervision is always considered synchronous (i.e., occurring in real time). The supervising practitioner is legally responsible for the episode of care and for the purposes of this survey, supervision does not include interactions outside of patient care episodes.  

End of Block: Block 2 - Supervision definitions

Start of Block: Block 3 - Placement

instruction Please answer the following questions.

placement What is your clinical placement? [Check all that apply]

- Primary care
- Outpatient mental health

| 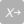 |
| --- |

discipline What is your clinical discipline? [Check all that apply]

- Internal Medicine
- Family Medicine
- Nursing
- Psychiatry
- Psychology

status Are you a **current** VA health professions trainee (resident, intern, fellow, or student)?

- Yes
- No

End of Block: Block 3 - Placement

Start of Block: Block 4 - Modalities

context_1 When answering the following questions, please consider your VA supervision experiences (both in-person and virtually) over the past year.

modality Which of the following modalities of supervision have you and your VA supervising practitioner(s) used? [Check all that apply]

- **In-person supervision** (meetings occur in person, face-to-face)
- **Video virtual supervision** (meetings occur virtually, using a method that allows video viewing of each other)
- **Telephone virtual supervision** (meetings occur virtually, using a method that allows you to hear each other's voices but not see any video)

| 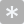 |
| --- |

proportion What proportion of your VA supervision occurred in the following modalities? [please use whole numbers; responses should total to 100]

**In-person supervision** (meetings occur in person, face-to-face) : _______

**Video virtual supervision** (meetings occur virtually, using a method that allows video viewing of each other) : _______

**Telephone virtual supervision** (meetings occur virtually, using a method that allows you to hear each other's voices but not see any video) : _______

Total : ________

End of Block: Block 4 - Modalities

Start of Block: Block 5 - Emergency Contact/Safety of Virtual Supervision

context_2 When answering the following questions, please consider your VA supervision experiences (both in-person and virtually) over the past year.

| 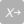 | 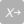 |
| --- | --- |

challenges_1 Have you experienced any of the following during your VA supervisory sessions? [Check all that apply]

- Internet connectivity/bandwidth issues
- Supervision sessions interrupted by non-work environmental distractions
- Software applications (including instant messaging applications) freezing, locking, not launching, etc.
- Hardware issues (e.g., laptops)
- Inability to find a private space for virtual supervision
- Reliable access to VA's network
- Other (please specify): __________________________________________________
- ⊗None of the above

| 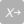 | 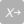 |
| --- | --- |

challenges_2 How disruptive were each of the following to your VA training experience?

|  | A little | Moderately | Very much |
| --- | --- | --- | --- |
| Internet connectivity/bandwidth issues |  |  |  |
| Supervision sessions interrupted by non-work environmental distractions |  |  |  |
| Software applications (including instant messaging applications) freezing, locking, not launching, etc. |  |  |  |
| Hardware issues (e.g., laptops) |  |  |  |
| Inability to find a private space for virtual supervision |  |  |  |
| Reliable access to VA's network |  |  |  |
| Other: ${challenges_1/ChoiceTextEntryValue/8} |  |  |  |

| Page Break |  |
| --- | --- |

context_3 When answering the following questions, please consider your VA supervision experiences (both in-person and virtually) over the past year.

| 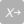 |
| --- |

contact_1 If you have contacted your VA supervising practitioner(s) outside of scheduled times, which of the following concerns were discussed? [Check all that apply]

- Personal issue/concern
- Patient care issue/concern
- Assistance with strategic decision-making related to professional issues (e.g., discussing job opportunities, career advice)
- Support for professional advancement (e.g., providing letters of recommendation, interview preparation)
- Ethical issues/concerns
- Concerns about colleagues or staff
- Other (please specify): __________________________________________________
- ⊗I have not contacted my VA supervising practitioner(s) outside of scheduled supervision times

contact_2 How available has your VA supervising practitioner(s) been to discuss the following:

|  | Not at all | A little | A moderate amount | A lot | Completely |
| --- | --- | --- | --- | --- | --- |
| Personal issue/concern |  |  |  |  |  |
| Patient care issue/concern |  |  |  |  |  |
| Assistance with strategic decision-making related to professional issues (e.g., discussing job opportunities, career advice) |  |  |  |  |  |
| Support for professional advancement (e.g., providing letters of recommendation, interview preparation) |  |  |  |  |  |
| Ethical issues/concerns |  |  |  |  |  |
| Concerns about colleagues or staff |  |  |  |  |  |
| Other: ${contact_1/ChoiceTextEntryValue/7} |  |  |  |  |  |

| Page Break |  |
| --- | --- |

context_4 When answering the following questions, please consider your VA supervision experiences (both in-person and virtually) over the past year.

emergency_prep To what extent has your VA supervision prepared you to address patient emergencies?

- Not at all
- A little
- A moderate amount
- A lot
- Completely

End of Block: Block 5 - Emergency Contact/Safety of Virtual Supervision

Start of Block: Block 6 - Competencies

comp_1 Please indicate the extent to which your VA supervision experiences over the past year have contributed your skill development in the following area: **Patient care**: providing patient-centered care that is compassionate, appropriate, and effective in the treatment of health problems and the promotion of health

- 0
- 1
- 2
- 3
- 4
- 5
- 6
- 7
- 8
- 9
- 10

| Page Break |  |
| --- | --- |

comp_2 Please indicate the extent to which your VA supervision experiences over the past year have contributed your skill development in the following area: **Knowledge for practice:** demonstrating knowledge of established and evolving biomedical, clinical, epidemiological and social-behavioral sciences, as well as the application of this knowledge to patient care

- 0
- 1
- 2
- 3
- 4
- 5
- 6
- 7
- 8
- 9
- 10

| Page Break |  |
| --- | --- |

comp_3 Please indicate the extent to which your VA supervision experiences over the past year have contributed your skill development in the following area: **Interpersonal and communication skills:** demonstrating interpersonal and communication skills that result in the effective exchange of information and collaboration with patients, their families, and health professionals

- 0
- 1
- 2
- 3
- 4
- 5
- 6
- 7
- 8
- 9
- 10

| Page Break |  |
| --- | --- |

comp_4 Please indicate the extent to which your VA supervision experiences over the past year have contributed your skill development in the following area: **Professionalism:** demonstrating a commitment to carrying out professional responsibilities, an adherence to ethical principles, and sensitivity to a diverse patient population

- 0
- 1
- 2
- 3
- 4
- 5
- 6
- 7
- 8
- 9
- 10

| Page Break |  |
| --- | --- |

comp_5 Please indicate the extent to which your VA supervision experiences over the past year have contributed your skill development in the following area: **Practice-based learning and improvement:**comprehending relevant information and a commitment to lifelong learning

- 0
- 1
- 2
- 3
- 4
- 5
- 6
- 7
- 8
- 9
- 10

| Page Break |  |
| --- | --- |

comp_6 Please indicate the extent to which your VA supervision experiences over the past year have contributed your skill development in the following area: **Systems-based practice:**demonstrating an awareness of and responsiveness to the larger context and system of health care, as well as the ability to call effectively on other resources in the system to provide optimal health care

- 0
- 1
- 2
- 3
- 4
- 5
- 6
- 7
- 8
- 9
- 10

End of Block: Block 6 - Competencies

Start of Block: Block 7 - Subjective Assessment of Supervision Experiences/Preferences

context_5 When answering the following questions, please consider your VA supervision experiences (both in-person and virtually) over the past year.

supervision Please indicate the extent to which you agree or disagree with the following statements.

|  | Completely disagree | Somewhat disagree | Neither agree nor disagree | Somewhat agree | Completely agree |
| --- | --- | --- | --- | --- | --- |
| Overall, I am satisfied with my supervision at VA. |  |  |  |  |  |
| My VA supervising practitioner(s) have an accurate view of my performance. |  |  |  |  |  |
| I receive useful feedback about my performance within my VA supervision. |  |  |  |  |  |
| My VA supervision allows for opportunities for professional modelling by my supervising practitioner(s). |  |  |  |  |  |
| My VA supervision allows for opportunities to discuss topics related to professional development. |  |  |  |  |  |
| I am satisfied with the frequency that I meet with my VA supervising practitioner(s). |  |  |  |  |  |
| I am satisfied with the amount of time I spend with my VA supervising practitioner(s). |  |  |  |  |  |

| Page Break |  |
| --- | --- |

context_6 When answering the following questions, please consider your VA supervision experiences (both in-person and virtually) over the past year.

remote_1 Overall, to what extent do you feel virtual supervision positively contributes to your VA training?

- Not at all
- A little
- A moderate amount
- A lot
- Completely

remote_2 Overall, to what extent do you feel virtual supervision negatively interferes with your VA training?

- Not at all
- A little
- A moderate amount
- A lot
- Completely

| Page Break |  |
| --- | --- |

context_7 When answering the following questions, please consider your VA supervision experiences (both in-person and virtually) over the past year.

remote_3 Please indicate your level of agreement with the following statement: The combination of in-person and virtual supervision that I receive in VA is a good match to my learning needs.

- Completely disagree
- Somewhat disagree
- Neither disagree nor agree
- Somewhat agree
- Completely agree

remote_4 Considering your VA training experience, what amount of virtual supervision would you prefer?

- Prefer fully virtual (100% virtual)
- Prefer mostly virtual (75% virtual)
- Prefer equal virtual/in-person (50% virtual)
- Prefer mostly in-person (25% virtual)
- Prefer fully in-person (0% virtual)

remote_5 Is virtual supervision available in your VA training experience?

- Yes
- No
- Unsure

remote_6 Are you interested in receiving virtual supervision in your VA training experience?

- Yes
- No
- Unsure

End of Block: Block 7 - Subjective Assessment of Supervision Experiences/Preferences

Start of Block: Block 8 - Trainee-Supervisor Relationship

context_8 When answering the following questions, please consider your VA supervision experiences (both in-person and virtually) over the past year.

relationship Please indicate the extent to which you agree or disagree with the following statements.

|  | Completely disagree | Somewhat disagree | Neither agree nor disagree | Somewhat agree | Completely agree |
| --- | --- | --- | --- | --- | --- |
| My VA supervising practitioner(s) and I effectively collaborate to support patient care decisions. |  |  |  |  |  |
| I feel that my VA supervising practitioner(s) have my best interests in mind. |  |  |  |  |  |
| I feel respected and heard by my VA supervising practitioner(s). |  |  |  |  |  |
| I feel comfortable working with my VA supervising practitioner(s). |  |  |  |  |  |
| My VA supervising practitioner(s) trust my decisions related to patient care. |  |  |  |  |  |

End of Block: Block 8 - Trainee-Supervisor Relationship

Start of Block: Block 9 - Demographics

demographics Lastly, we would like to ask you some questions about your background. As a reminder, responses to this survey will be kept confidential. You will not be identified individually in any reporting. All responses will be reported in aggregate.

tenure How many months have you been a VA health professions trainee (resident, intern, fellow, or student)?

- Less than 6 months
- 6-12 months
- 13-18 months
- More than 18 months

age What is your age?

▼ 18-24 ... 45 and up

gender Which term do you use to identify your gender?

- Woman
- Man
- Non-binary
- Other/Prefer to self-describe
- Prefer not to say

ethnicity Are you of Hispanic, Latino, or of Spanish origin?

- Yes
- No

race What term(s) would you use to describe your race? [Check all that apply]

- American Indian or Alaska Native
- Asian
- Black or African American
- Native Hawaiian or Other Pacific Islander
- White
- Other

End of Block: Block 9 - Demographics

Start of Block: Block 10 - Request for feedback

feedback What feedback do you have to OAA about the role of virtual supervision in VA?

________________________________________________________________

________________________________________________________________

________________________________________________________________

________________________________________________________________

________________________________________________________________

End of Block: Block 10 - Request for feedback
